# In vitro propagation of Indonesian stevia (*Stevia rebaudiana*) genotype using axenic nodal segments

**DOI:** 10.1186/s13104-024-06703-0

**Published:** 2024-02-05

**Authors:** Nurul Jadid, Suci Anggraeni, Muhammad Rifqi Nur Ramadani, Mutiara Arieny, Faisol Mas’ud

**Affiliations:** 1 Department of Biology, Institut Teknologi Sepuluh Nopember, Surabaya, 60111 Indonesia; 2 Department of Aquatic Resources Management, Faculty of Fisheries, Islamic University of Lamongan, Lamongan, 62211 Indonesia

**Keywords:** Callogenesis, Micropropagation, *Stevia rebaudiana*, Organogenesis, Plant growth regulators

## Abstract

**Objective:**

The high industrial demand for Stevia cultivation (*Stevia rebaudiana*) has increased due to its high stevioside content derived from the leaves. However, the low germination rate makes the cultivation of the plant become the main obstacle. Therefore, an efficient cultivation technique is required. This present work aims to analyze the effect of five combinations of Kinetin (Kin) and benzyladenine (BA) on stevia micropropagation using nodal segment explants.

**Results:**

The micropropagation of stevia was performed using Murashige and Skoog (MS) medium supplemented with BA and Kin. We analyzed different organogenesis and callogenesis responses. In addition, the number of shoots and root formed during in vitro culture were also observed. Our results demonstrated that all treatments with Kin, both alone and in combination with BA, resulted in the development of callus on all nodal segment explants. Explants treated in MS with 1 mg L^−1^ BA exhibited the best average of shoot number (36.27). In contrast, the treatment without PGR resulted in the best root formation (2.6). The overall results suggested that different combination of BA and Kin resulted in distinct organogenesis responses, where 1 mg L^−1^ of BA was potentially used for boosting the number of shoots in micropropagation of stevia accession Mini.

**Supplementary Information:**

The online version contains supplementary material available at 10.1186/s13104-024-06703-0.

## Introduction

The study on tropical medicinal plants is of particular interest to biotechnology-based industries. A large portion of pharmaceutical compounds have been investigated and produced [1]. Stevia (*Stevia rebaudiana*) is a food-flavoring and medicinal herb plant indigenous to Paraguay. This perennial plant is a member of the Compositae family. Stevia leaves are known to be sweeter than sugarcane. This is mainly due to the glycosides content [2]. Stevia leaf glycosides are calorie-free and have a nearly zero glycemic index, making them suitable for individuals with diabetes and those aiming to lose weight [3, 4]. Stevia sugar is widely used in the food and beverage industries as well as for its antibacterial and antioxidant properties. The sweetness derived from Stevioside is not metabolized in the body, making it highly recommended for individuals with diabetes, hypertension, obesity, and fungal infections [5, 6]. The utilization of stevia as a sweetener has been well-established in advanced countries such as the United States and Japan. In Japan, 5.6% of marketed sugar is stevia sugar, known as “sutebia” [7].

The widespread application of stevia in various industries has created promising opportunities for stevia cultivation. However, stevia cultivation faces challenges in its propagation. Low germination rate makes the cultivation of the plant become the main obstacle [8]. Conventionally, the propagation of stevia uses stem cuttings. Nevertheless, it requires a lot of mother plants as main sources, making large-scale cultivation inefficient [9]. Therefore, finding a rapid and efficient method of propagation is necessarily needed. Plant in vitro propagation might be an alternative way to accelerate plant cultivation.

In vitro propagation takes advantage of the totipotent properties of the plant cells to self-regenerate into many genetically identical new plants [10]. This method requires appropriate culture media supplemented with plant growth regulators (PGRs). Some studies showed that different types of PGRs affected the ability of shoot regeneration. Cytokinin-based PGRs have been known to induce shoot formation and proliferation [11]. Several studies used 6-Benzyladenine (BA) and Kinetin (Kin) to mass propagate stevia via tissue culture technique [12, 13]. However, each plant genotype develops different responses against environmental conditions [14]. Moreover, different genetic profiles of the plants among plant species also resulted in a distinct in vitro growth performance of the plant [15, 16].

In some stevia tissue culture studies, a variety of PGRs have been used to promote shoot proliferation. For instance, 6-benzylamino purine (BAP), Kin, and BA [17, 18]. Silver nanoparticles (AgNps) in plant in vitro micropropagation was also recently reported to accelerate shoot proliferation [19, 20]. Interestingly, plant growth responses during in vitro culture may vary depending on the PGRs concentration [21, 22]. Some stevia genotypes have been cultivated in Indonesia. It includes stevia accession “green,” “Jumbo,” “purple,” “yellow,” and “mini.” This present work was conducted to investigate the influence of BA and Kin on stevia (genotype mini) tissue culture.

## Materials and methods

### Sterilization of plant explants and establishment of culture medium

The stevia (*S. rebaudiana*) accession Mini was obtained from the Indonesian Sweetener and Fiber Crops Research Institute. Some uniform nodal segments were collected to be used further as explants. Before being subjected to surface sterilization, the nodal segments were cleaned using continuous tap water for 30 min. Subsequently, 70% ethanol (EtOH) was used to immerse the explants for 1 min. The explants were then surface sterilized by submerging in a 1.5% sodiumhypochlorite (NaOCl) for 5 min. Subsequently, the nodal segments were washed four times using sterile aquaest to eliminate sterilant agent traces.

The solid MS culture medium was prepared by mixing 4.4 g/L MS media (PhytoTech Lab^®^) and 30 g/L sucrose (Duchefa Biochemie, Netherlands). The culture medium was solidified with 8.2 g/L gelrite powder (PhytoTech Lab^®^). A series of PGRs concentrations were applied to the MS medium. The concentration of BA ranged from 0 to 2 mg L^−1^, whereas the concentration of Kin was 0, 2, 4, and 8 mg L^−1^. A 5.7–5.8 pH adjustment was made to the medium before being poured (25 ml) into sterile containers. Finally, the prepared culture medium was autoclaved for 20 min at 121 °C.

### In vitro inoculation and growth conditions

The previously sterilized nodal explants were then inoculated into an MS solid medium without PGRs. They were grown in a culture room under 40 W of cool white fluorescent light and at 25 ± 2 °C for ten weeks. We used the axenic nodal segments obtained from the previous culture as secondary explants for this present work. The axenic nodal segments were placed into an MS medium containing different combinations of BA and Kin. All treatments were incubated in a grow room at 25 ± 2 °C under 40W of cool white fluorescent, 16/8 h (light/dark) photoperiod for ten weeks. Each treatment consisted of five replications.

### Plant growth measurement and data analysis

The growth response was determined based on shoot formation and callogenic frequency [21]. The equations used in this study were as follows:

1 $$Shoot \,formation\,frequency =\frac{ Number \,of \,explants \,forming \,shoots}{Total \,number \,of \,explants}x 100$$

2 $$Callogenic \,frequency =\frac{ Number \,of \,explants \,forming \,callus}{Total \,number \,of \,explants}x 100$$

Mean number of shoots and roots formed during the incubation period was also measured and statistically analyzed using two-way anaylsis of variance (Minitab 19), followed by the Tukey post-hoc test.

## Results

### Organogenesis and callogenesis responses

Plant Growth Regulators (PGRs) are non-nutrient organic compounds functioning at low concentrations to accelerate or inhibit plant growth and development processes. We observed that different concentrations of BA and Kin resulted in distinct organogenesis and callogenesis responses (Table 1). Our results demonstrated that almost all of the nodal segments grown on MS medium with different PGRs combinations formed shoot. However, their frequency of shoot formation varied and ranged from 45 to 100% (Table 1). Notably, treatment with 1 mg L^−1^ BA and 8 mg L^−1^ Kin exhibited the lowest shoot formation. Meanwhile, treatment with BA alone (0.5; 1; 1.5, and 2 mg L^−1^) and with BA and a low Kin concentration (2 mg L^−1^) led to the induction of a significant shoot formation.

**Table 1:** Percentage of explants showing organogenesis and callogenesis responses in *S. rebaudiana* genotype Mini after 10 weeks of culture

Combination of plant growt regulators		Shoot formation frequency (%)	Callus formation frequency (%)
BA (mg L^−1^)	Kin (mg L^−1^)		
0	0	100	0
0.5	0	100	0
1	0	100	0
1,5	0	100	0
2	0	100	0
0	2	90	100
0,5	2	100	100
1	2	90	100
1,5	2	100	100
2	2	65	100
0	4	85	100
0.5	4	90	100
1	4	60	100
1.5	4	65	100
2	4	60	100
0	6	75	100
0.5	6	75	100
1	6	50	100
1.5	6	60	100
2	6	85	100
0	8	95	100
0.5	8	75	100
1	8	45	100
1.5	8	75	100
2	8	85	100

**Fig. 1:**
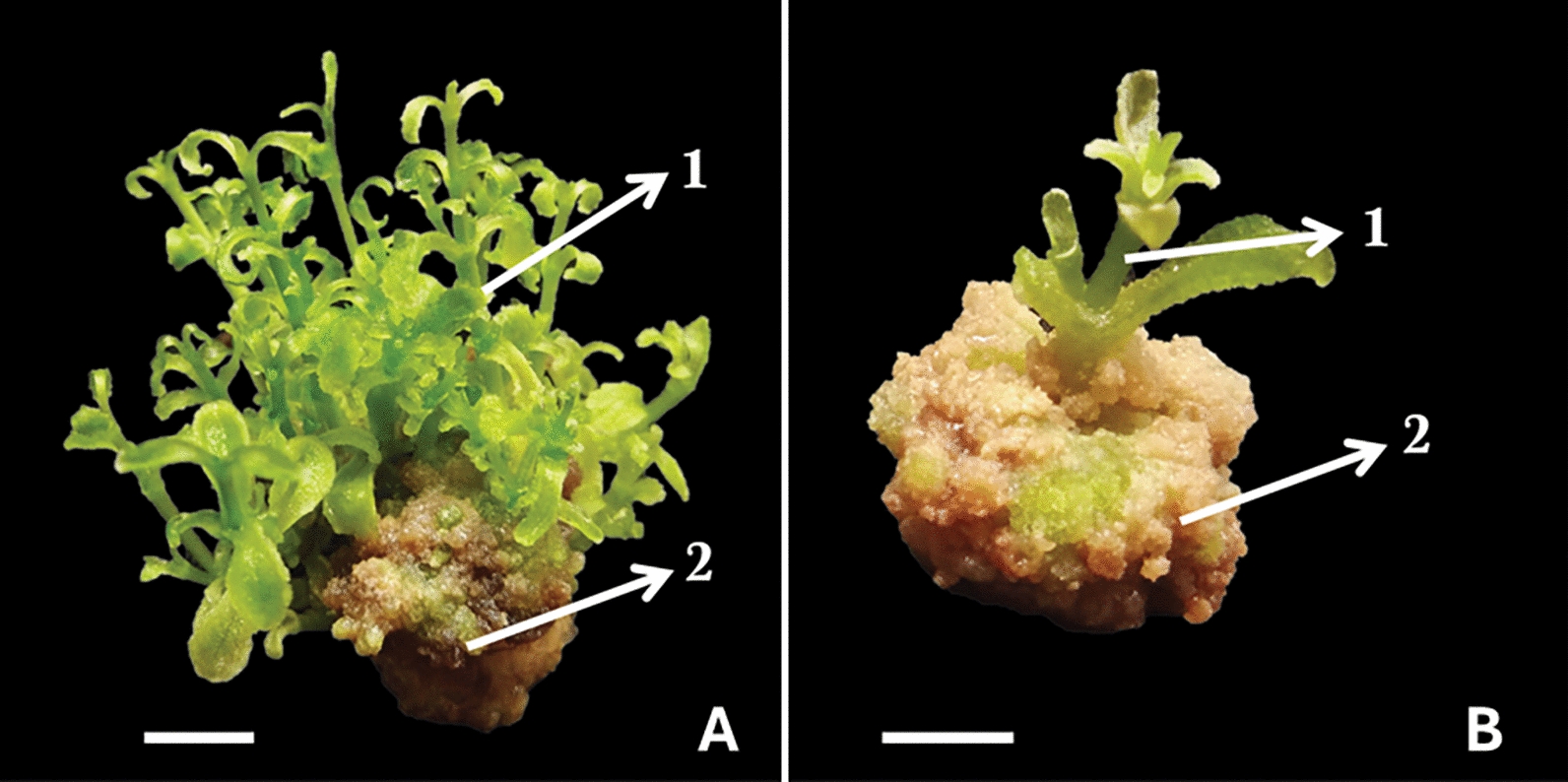
Organogenesis and callogenesis respons of *S. rebaudiana* after ten weeks of culture. **A**. Axenic nodal segment of *S. rebaudiana* treated with 0.5 mg L^−1^ BA + 2 mg L^−1^ Kin; **B**. 0.5 mg L^−1^ BA + 4 mg L^−1^ Kin. White arrows indicate: (1) shoot; (2) callus (White bar = 0.5 cm)

Further analysis showed that explants responded to the PGRs tested in the form of callus. Interestingly, explants developed callus in all treatments using low and high Kin concentrations. In contrast, an absence of callogenesis was resulted in treatment without the addition of Kin (Table 1). Our morphological observation also demonstrated that the callus formed in this study was compact and brownish (Fig. 1). Our findings imply that BA is crucial to enhancing shoot formation, whereas Kin promoted callus formation in stevia tissue culture.

### Shoot multiplication in stevia tissue culture

**Fig. 2:**
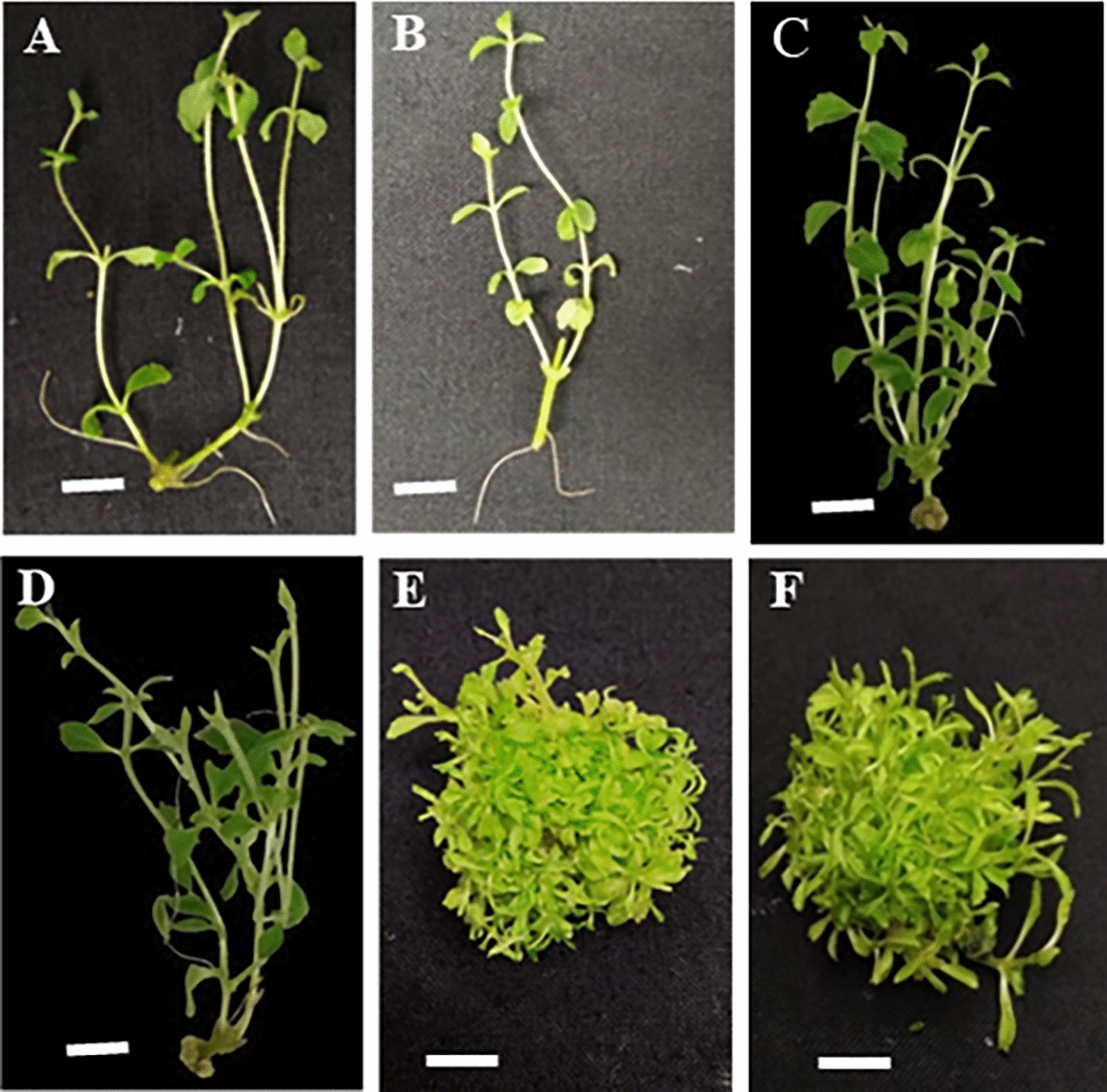
Shoot formation in *Stevia Rebaudiana* tissue culture after ten weeks of treatment. Shoot derived from explant treated in MS with no PGRs applied (**A**, **B**); 0.5 mg L^−1^ BA (**C**, **D**); 1 mg L^−1^ BA (**E**, **F**) (White bar = 0.5 cm)

Shoot formation is the primary goal of plant in vitro propagation. Appropriate and optimal concentrations of PGRs potentially induce the formation of shoots. A two-way ANOVA analysis revealed that combining Kin and BA with different concentrations significantly affected the number of shoots (P value < 0.05). Treatment with BA alone (1 mg L^−1^) generated the most significant number of shoots (36.27 shoots per explant) (Fig. 2). In contrast, a combination of 1 mg L^−1^ BA and 8 mg L^−1^ Kin resulted in the lowest number of shoots (0.71) (Table 2). Notably, an absence of BA resulted in low shoot formations (Table 2). Therefore, it might suggest that BA induces shoot multiplication.

**Table 2:** Effect of Kin and BA combinations in shoot proliferation of the stevia tissue culture after 10 weeks of culture

Combination of PGRs		Average number of shoots	Combination of PGRs		Average number of shoots
BA (mg L^−1^)	Kin (mg L^−1^)		BA (mg L^−1^)	Kin (mg L^−1^)	
0	0	2.06 ± 0.24^e^	1,5	4	1.2 ± 0.42^e^
0,5	0	2.36 ± 0.92^e^	2	4	1.14 ± 0.38^e^
1	0	36.27 ± 6.36^a^	0	6	1.75 ± 0.35^e^
1,5	0	17.46 ± 3.69^c^	0,5	6	2.07 ± 0.27^e^
2	0	26.75 ± 5.83^b^	1	6	1.13 ± 0.35^e^
0	2	1.93 ± 0.27^e^	1,5	6	1.22 ± 0.44^e^
0,5	2	8.79 ± 2.91^d^	2	6	1.67 ± 0.49^e^
1	2	3.33 ± 1.67^e^	0	8	1.88 ± 0.33^e^
1,5	2	7.36 ± 1.57^d^	0,5	8	1.10 ± 0.32^e^
2	2	1.58 ± 0.51^e^	1	8	0.71 ± 0.49^e^
0	4	1.92 ± 0.28^e^	1,5	8	2.08 ± 0.28^e^
0,5	4	1.93 ± 0.26^e^	2	8	1.85 ± 0.38^e^
1	4	1.11 ± 0.33^e^			

^*^Different letters mean statistically significant difference between all treatments at 0.05 level

### Effect of BA and KIN combination on root formation in stevia tissue culture

**Fig. 3:**
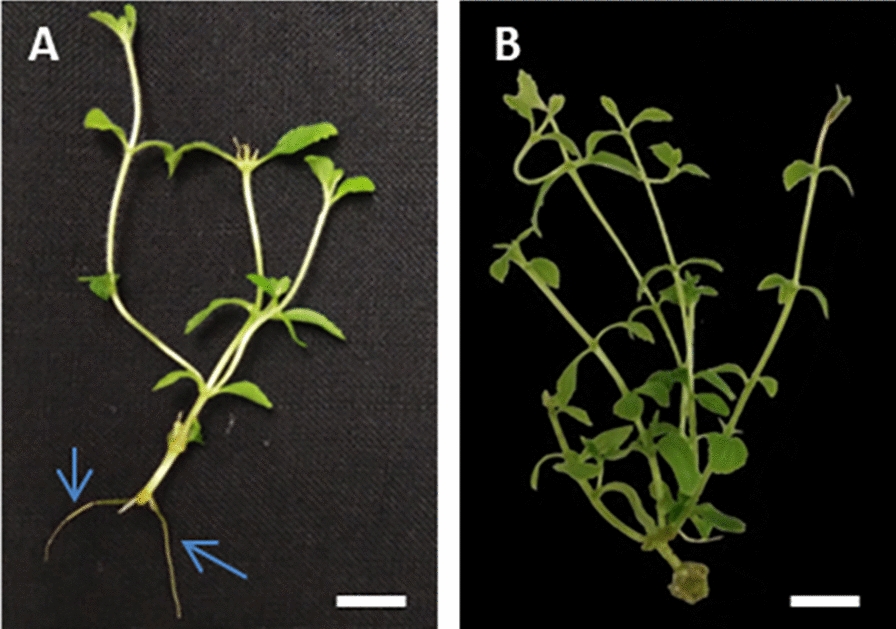
Root formation in *Stevia Rebaudiana* tissue culture after 10 weeks of culture. Root (blue arrows) derived from explant treated in MS 0 (**A**); MS with 0.5 mg L^−1^ BA (**B**). (White bar = 0.5 cm)

Root formation is essential for preparing the plantlets before being transferred to the greenhouse. In this study, we reported that several explants responded to root formation (Additional file 1: Table S1). Our data showed that the interaction between BA and Kin significantly affected the number of roots per explant (p-value < 0.05). Interestingly, explants treated with MS 0, without PGRs addition had highest number of roots (2.64 roots per explants) (Additional file 1: Table S1). A low level of cytokinin might help the development of roots in stevia. This study detected few root formations when explants were treated in MS with 0.5 mg L^−1^ BA. Meanwhile, greater BA and Kin concentrations resulted in zero root formations (Fig. 3).

## Discussion

Plant organogenesis response is a critical parameter in plant in vitro micropropagation studies. It is tightly correlated with the application of PGR. The latter directs plant growth and the differentiation of plant cells and tissue during regeneration. This present study used two cytokinin-based PGRs, the 6-Benzyladenine (BA) and Kinetin (Kin). Previous studies showed that adding BA and Kin resulted in good shoot proliferation in *Thuarea involuta* and *Hyoscyamus niger* L. [23, 24]. Another study also reported that combining BA and Kin can synergistically promote shoot regeneration in *Lagenaria siceraria* [25]. Even though both BA and Kin give different plant organogenesis responses. Our data demonstrated that BA significantly induced shoot formation in stevia tissue culture, compared to Kin. Our findings support previous studies on stevia in vitro culture [17] [26, 27].

Callogenesis is another effect that commonly appears during a plant in vitro culture. It describes the development of an amorphous and disorganized mass of cells forming on plant explants’ surface [28]. It is worth noting that all Kin concentrations added in the MS medium could induce callus formation. It was described that the use of 3–5 mg L^−1^ of Kin results in callus formation [29]. The regulation of cytokinin in promoting callus formation is less clear than those promoted by auxins. However, it is believed that type B *Arabidopsis Response Regulators* (ARRs) mediate callus induction [30]. It was also reported that cytokinins induce plant division, leading to the formation of undifferentiated callus [31].

In this study, we reported that 1 mg L^−1^ BA induced a significant number of stevia shoots. It was also reported that BA was more effective for stevia shoot multiplication [12, 32]. Several studies also described the effectiveness of BA to induce shoot multiplication in other species such as *Kaempferia parviflora*, *Dalbergia nigra*, and *Cordia subcordata* [33–35]. BA and Kin are known as cytokinin-based PGR, which positively promotes shoot multiplication in plant tissue culture studies [36]. Basically, cytokinins stimulate plant cytokinesis [37]. Benzyladenine (BA) significantly shortened the S phase period during the cell cycle (from G2 to mitosis, the DNA and protein synthesis stages of cell division). It was postulated that cytokinins promote cell division in plant tissue culture by accelerating the transition from G2 to mitosis. In addition, cytokinin also regulates the plant growth-related protein synthesis needed for mitosis [38].

The application of cytokinin in this study apparently inhibit root formation. We noticed that no root initiation appeared in the explants treated with cytokinin, both Kin and BA. Nevertheless, we observed low numbers of the root has been initially formed in explants grown in zero Kin and BA (Additional file 1: Table S1). Our data consistent with the previous study stated that root growth reduction arose when plants received cytokinin application in *Arabidopsis thaliana* [39]. Exogenous cytokinin resulted in a reduction of meristem size in root apical meristem [40]. Other studies also reported that auxin and cytokinin demonstrated an antagonistic effect on root formation [41]. Auxin functions in promoting lateral root formation, while cytokinin appears to inhibit it [42, 43].

In summary, our findings suggest that nodal segment of *S. rebaudiana* served as potential explant to shoot micropropagate the plant. In addition, we noticed that type and concentration of the cytokinin influence shoot proliferation of the plant, where benzyl adenine at 1 mg L^−1^ served as optimum concentration. Further studies should be conducted to induce root formation of the plants for providing a complete cycle of in vitro propagation of *S. rebaudiana*. In this case, auxin-supplemented media might potentially increase the number of roots. Altogether, our findings might serve as an alternative method to conserve and proliferate Indonesian stevia genotype.

### Supplementary Information

**Additional file 1: Table S1.** Effect of Kin and BA combinations in root proliferation of the stevia tissue culture after ten weeks of treatment.

## Data Availability

All data used in this study are included in this published article.

## Abbreviations

ARRs: *Arabidopsis Response Regulators*
BA: Benzyladenine
EtOH: Ethanol
Kin: Kinetin
MS: Murashige and Skoog
NaOCl: Natrium hypochlorite
PGRs: Plant growth regulators
