# Supplementary material for: In vitro propagation of Indonesian stevia (Stevia rebaudiana) genotype using axenic nodal segments
Source: BMC Res Notes. 2024 Feb 5;17:45. doi: 10.1186/s13104-024-06703-0 (PMC10840238; doi:10.1186/s13104-024-06703-0)
Supplement: Supplementary file 1 — Additional file 1: Table S1. Effect of Kin and BA combinations in root proliferation of the stevia tissue culture after ten weeks of treatment. [file 13104_2024_6703_MOESM1_ESM.docx]

**Additional file 1**

**Table S1. Effect of Kin and BA combinations in root proliferation of the stevia tissue culture after ten weeks of treatment**

| Combination of PGRs | | Average number of roots | Combination of PGRs | | Average number of roots |
| --- | --- | --- | --- | --- | --- |
| BA  (mg L^-1^) | Kin  (mg L^-1^) |  | BA  (mg L^-1^) | Kin  (mg L^-1^) |  |
| 0 | 0 | 2.64 ± 0.92^a^ | 1,5 | 4 | 0.0^b^ |
| 0,5 | 0 | 0,15 ± 0.02^b^ | 2 | 4 | 0.0^b^ |
| 1 | 0 | 0.0^b^ | 0 | 6 | 0.0^b^ |
| 1,5 | 0 | 0.0^b^ | 0,5 | 6 | 0.0^b^ |
| 2 | 0 | 0.0^b^ | 1 | 6 | 0.0^b^ |
| 0 | 2 | 0.0^b^ | 1,5 | 6 | 0.0^b^ |
| 0,5 | 2 | 0.0^b^ | 2 | 6 | 0.0^b^ |
| 1 | 2 | 0.0^b^ | 0 | 8 | 0.0^b^ |
| 1,5 | 2 | 0.0^b^ | 0,5 | 8 | 0.0^b^ |
| 2 | 2 | 0.0^b^ | 1 | 8 | 0.0^b^ |
| 0 | 4 | 0.0^b^ | 1,5 | 8 | 0.0^b^ |
| 0,5 | 4 | 0.0^b^ | 2 | 8 | 0.0^b^ |
| 1 | 4 | 0.0^b^ |  |  |  |

* Different letters mean statistically significant difference between all treatments at 0.05 level.
